# Supplementary material for: Mechanical properties of a biodegradable self-expandable polydioxanone monofilament stent: In vitro force relaxation and its clinical relevance
Source: PLoS One. 2020 Jul 8;15(7):e0235842. doi: 10.1371/journal.pone.0235842 (PMC7343154; doi:10.1371/journal.pone.0235842)
Supplement: S1 File — (PDF) [file pone.0235842.s001.pdf]

For our purposes, we used the two equations published by Zahora *et al.* [1]: Equation (e1) (published by Zahora *et al.* [1] as Equation (15) on page S118) showing the relationship between radial pressure p_R and axial force F_Z

$$p_R = 4\pi \frac{F_Z}{kl^2 \sin^2 \alpha} \tag{e1}$$

and Equation (e2) (published by Zahora *et al.* [1] as Equation (14) on page S118) showing the relationship between radial pressure p_R and radial force F_R

$$p_R = \frac{F_R}{kl^2 (\sin \alpha)(\cos \alpha)} \tag{e2}$$

where α is the pitch angle, l is the length of a wire of a single turn of a single helix of the stent and k is the number of turns of a single helix of the stent. The relationship among the α, l, and k is shown in Fig f1.

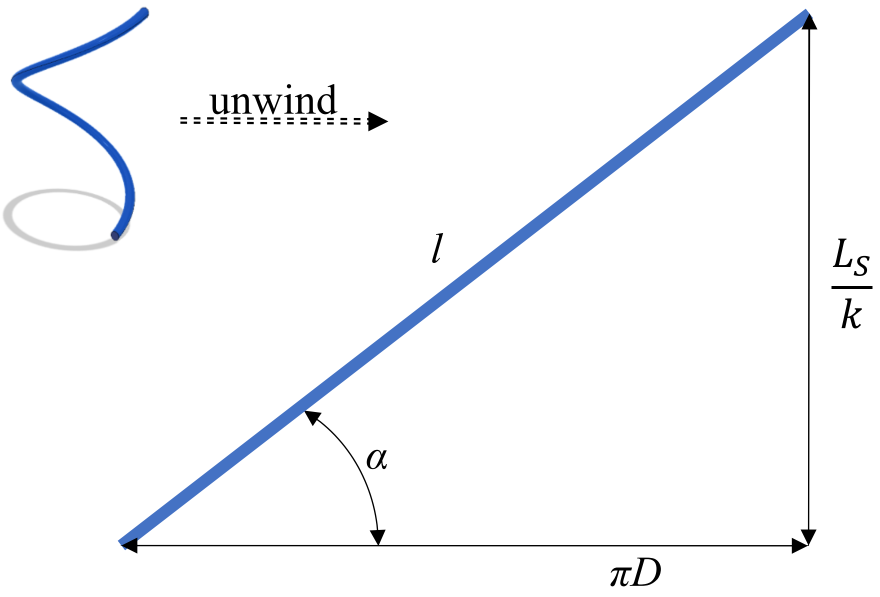

Fig f1. Unwound single turn of a single helix of the stent. α is the pitch angle, l is the length of a wire of a single turn of a single helix of the stent, k is the number of turns of a single helix of the stent, L_S is the measured instantaneous total length of the stent, and πD is the instantaneous circumference of the stent (D is the instantaneous diameter of the stent).

Now, we easily derive the following relationship:

$$\frac{L_S}{k} = l \sin \alpha \tag{e3}$$

The parameter k can also be derived as the ratio of the nominal length of an unloaded stent, L_N to the length of a single turn of a single helix of the unloaded stent (helix length) L_H:

$$k = \frac{L_N}{L_H} \tag{e4}$$

Following substitution of Equations (e3) and (e4) into Equation (e1) gives:

$$p_R = 4\pi \frac{F_Z}{k\frac{L_S^2}{k^2}}$$

$$p_R = 4\pi k \frac{F_Z}{L_S^2}$$

$$p_R = 4\pi \frac{L_N}{L_H} \frac{F_Z}{L_S^2} \tag{e5}$$

Since the axial force denoted by Zahora *et al*. [1] as F_Z is the same axial force denoted as F_A ($F_A = F_Z$), using this substitution, we finally derive Equation (1) in our manuscript, which shows the relationship between radial pressure p_R and axial force F_A of the stent:

$$p_R = 4\pi \frac{L_N}{L_H} \frac{F_A}{L_S^2} \tag{1}$$

The following steps are only slightly more complicated. First, using Equations (e1) and (e2), we derive the relationship among radial force F_R, axial force F_Z, and the stent parameters k, l, and α:

$$4\pi \frac{F_Z}{kl^2 \sin^2 \alpha} = \frac{F_R}{kl^2 (\sin \alpha)(\cos \alpha)}$$

$$F_R = 4\pi F_Z \frac{\cos \alpha}{\sin \alpha} \tag{e6}$$

Next, as shown in Fig f1, we derive the relationship among $\cos\alpha$, the stent instantaneous circumference πD, and the length of a wire of a single turn of a single helix of the stent l:

$$\cos\alpha = \frac{\pi D}{l} \tag{e7}$$

We substitute Equations (e3) and (e7) into Equation (e6) as follows:

$$F_R = 4\pi F_Z \frac{\frac{\pi D}{l}}{\frac{L_S}{kl}}$$

$$F_R = 4\pi F_Z k \frac{\pi D}{L_S} \tag{e8}$$

Using the Pythagorean theorem, we also derive the relationship among the parameters l, πD, and L_S/k:

$$\pi D = \sqrt{l^2 - \frac{L_S^2}{k^2}} \tag{e9}$$

Similarly, we derive the among the parameters l, k, the nominal circumference of the unloaded stent πD_1, and the length of a single turn of a single helix of the unloaded stent (helix length) L_H:

$$l^2 = \pi^2 D_1^2 + \frac{L_N^2}{k^2} \tag{e10}$$

Using the successive substitution of Equations (e9), (e10), and (e4) into Equation (e8), we derive:

$$F_R = 4\pi F_Z \frac{k}{L_S} \sqrt{\pi^2 D_1^2 + \frac{L_N^2}{k^2} - \frac{L_S^2}{k^2}}$$

$$F_R = 4\pi F_Z \sqrt{\frac{k^2\pi^2 D_1^2 + L_N^2}{L_S^2} - 1}$$

$$F_R = 4\pi F_Z \sqrt{\frac{\left(\frac{L_N}{L_H}\right)^2 \pi^2 D_1^2 + L_N^2}{L_S^2} - 1}$$

$$F_R = 4\pi F_Z \sqrt{\frac{\left(\frac{\pi^2 D_1^2}{L_H^2} + 1\right) L_N^2}{L_S^2} - 1} \quad \text{(e11)}$$

And again, since the axial force denoted by Zahora *et al*. [1] as F_Z is the same axial force denoted as F_A ($F_A = F_Z$), using this substitution into equation (e11), we finally derive Equation (2) in our manuscript, which shows the relationship between radial force F_R and axial force F_A of the stent:

$$F_R = 4\pi F_A \sqrt{\frac{\left(\frac{\pi^2 D_1^2}{L_H^2} + 1\right) L_N^2}{L_S^2} - 1} \quad \text{(2)}$$

REFERENCES

1. Zahora J, Bezrouk A, Hanus J. Models of stents - Comparison and applications. Physiol Res. 2007;56: S115–S121.
