# Supplementary figures and images for: Mechanical properties of a biodegradable self-expandable polydioxanone monofilament stent: In vitro force relaxation and its clinical relevance
Source: PLoS One. 2020 Jul 8;15(7):e0235842. doi: 10.1371/journal.pone.0235842 (PMC7343154; doi:10.1371/journal.pone.0235842)

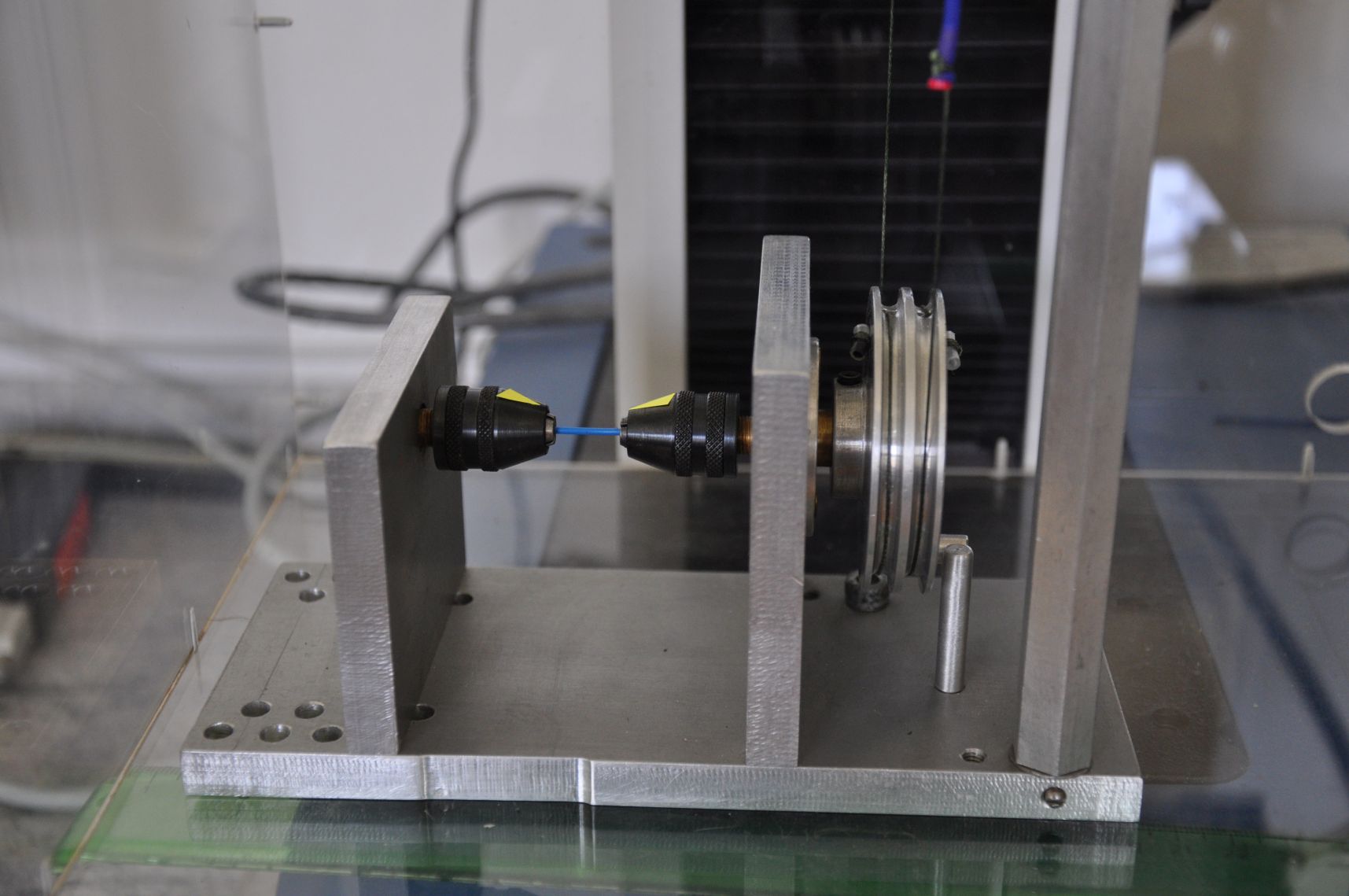

Supplement: S2 File — (ZIP) [file pone.0235842.s002.zip › Tool_Photo_and_Video/Tool_FrontDetail.jpg]

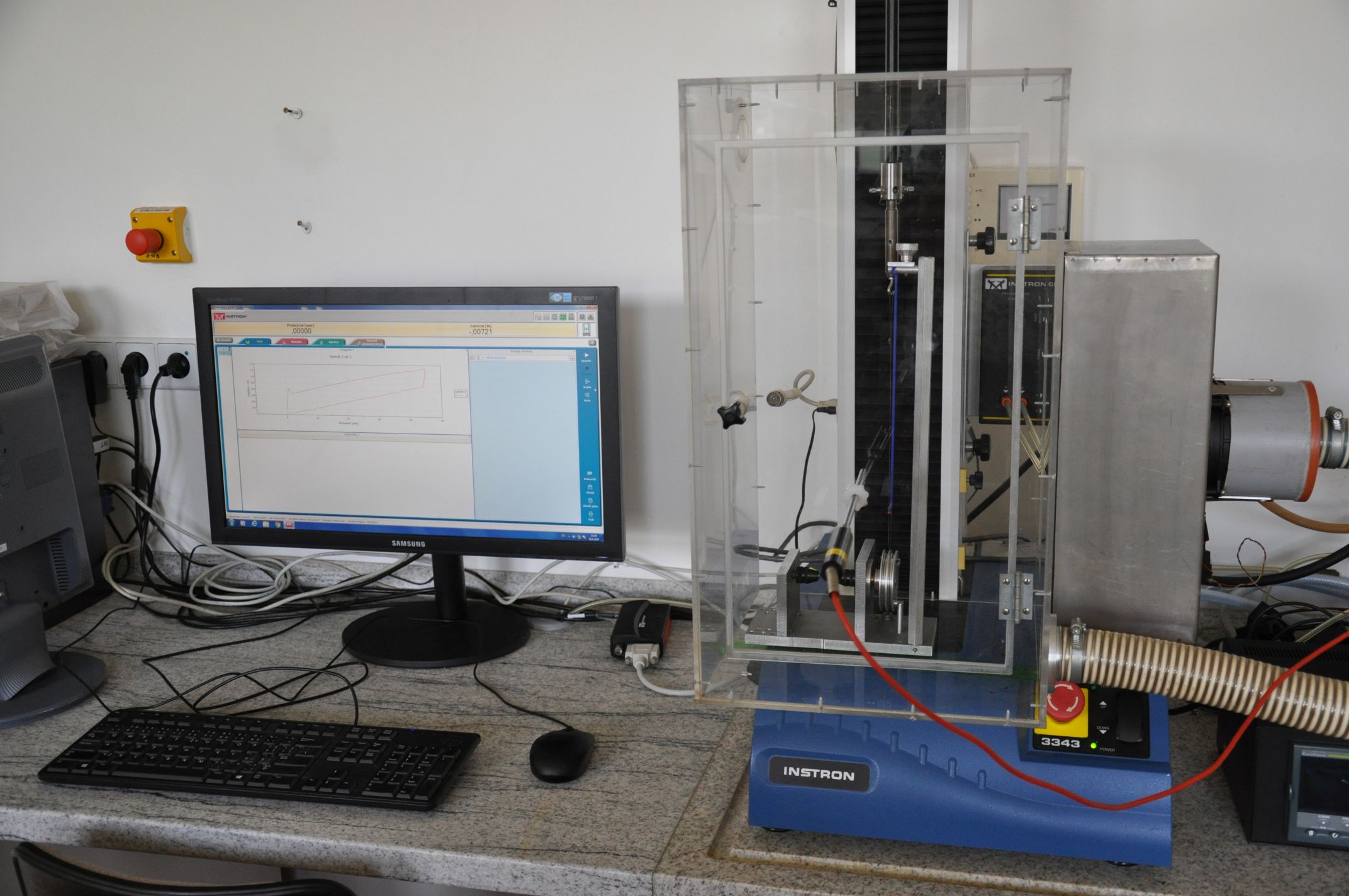

Supplement: S2 File — (ZIP) [file pone.0235842.s002.zip › Tool_Photo_and_Video/Tool_FrontOverview.jpg]

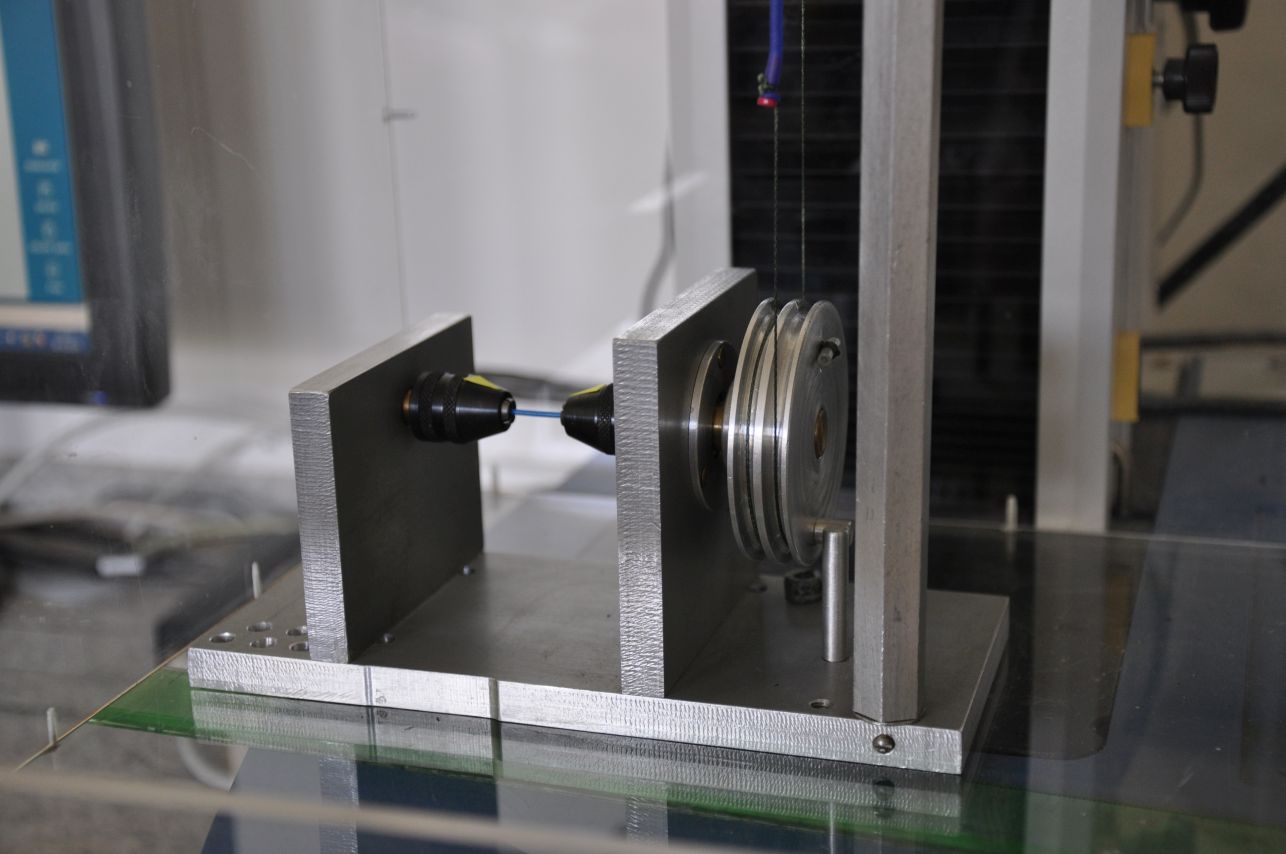

Supplement: S2 File — (ZIP) [file pone.0235842.s002.zip › Tool_Photo_and_Video/Tool_PerspectiveDetail_Pulley.jpg]

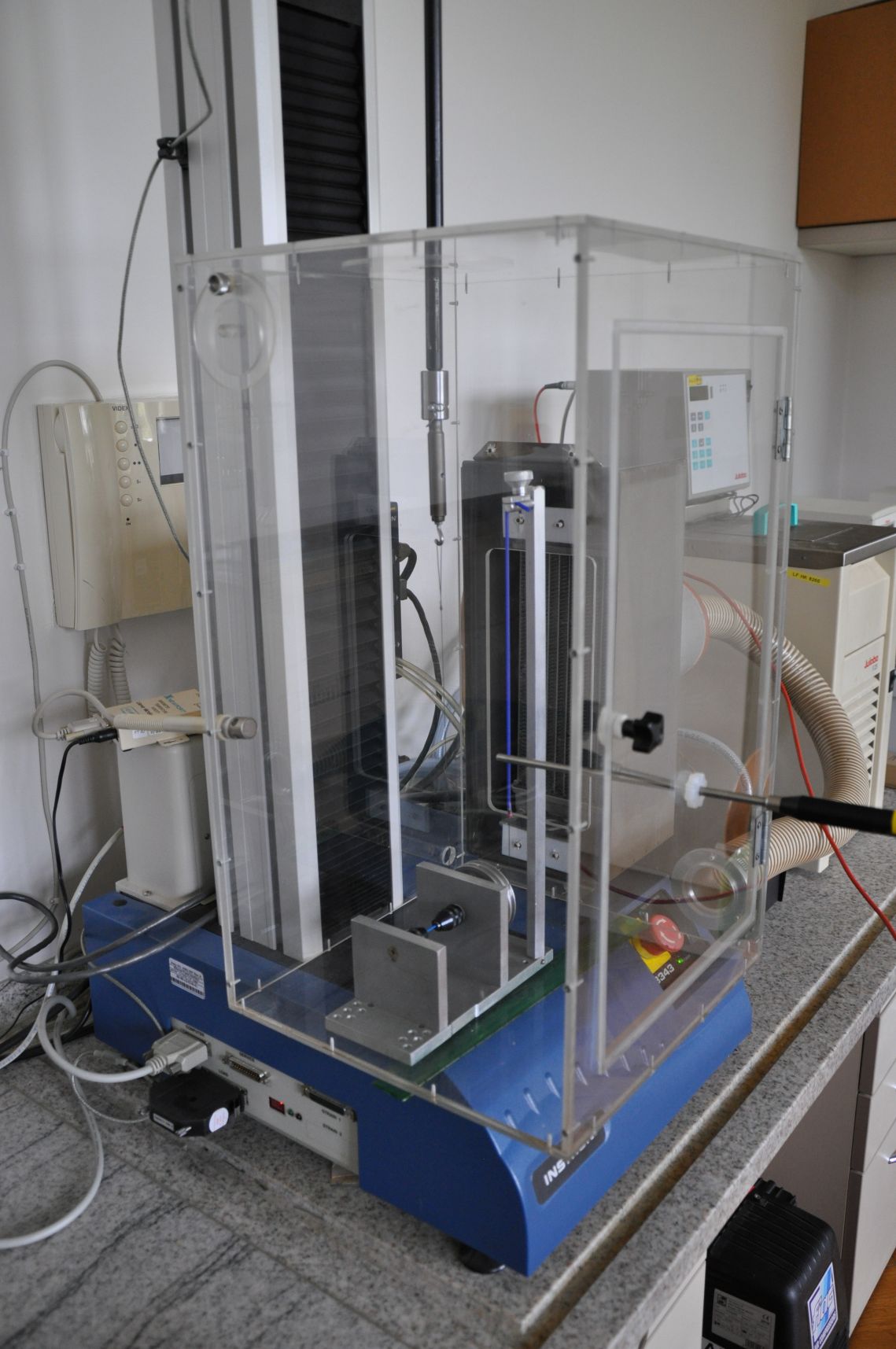

Supplement: S2 File — (ZIP) [file pone.0235842.s002.zip › Tool_Photo_and_Video/Tool_SideOverview.jpg]

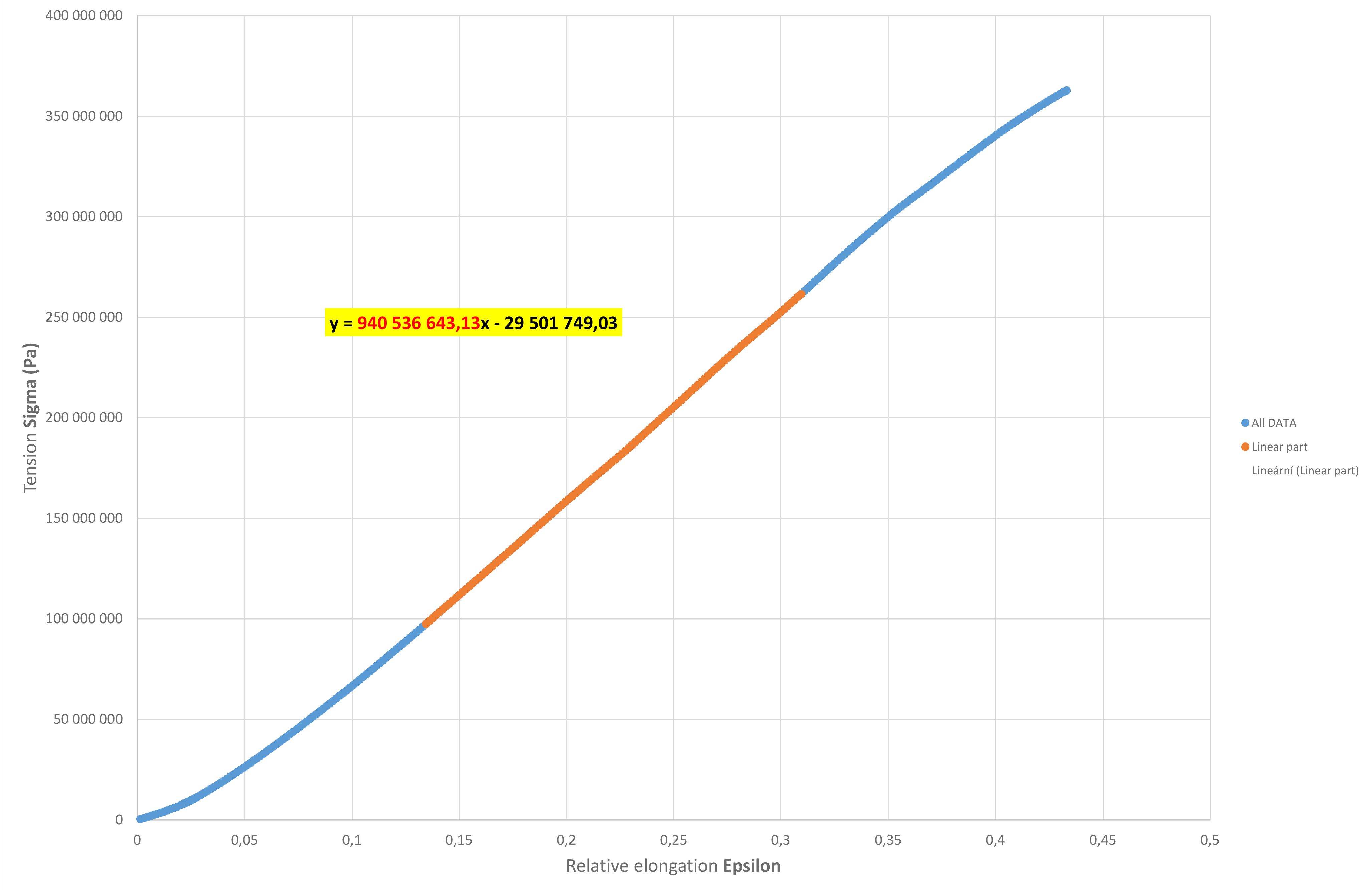

Supplement: S4 File — (ZIP) [file pone.0235842.s004.zip › Tensile_Data/Tensile1_Graph_Figure.tif]
